# Method Categorization of Stem Cell Therapy for Degenerative Osteoarthritis of the Knee: A Review

**DOI:** 10.3390/ijms222413323

**Published:** 2021-12-11

**Authors:** Jae Sun Lee, Dong Woo Shim, Kyung-Yil Kang, Dong-Sik Chae, Woo-Suk Lee

**Affiliations:** 1 Stem Cell Therapy Center, International St. Mary’s Hospital, College of Medicine, Catholic Kwandong University, Incheon 22711, Korea; jaesun127@gmail.com; 2 Department of Orthopedic Surgery, International St. Mary’s Hospital, College of Medicine, Catholic Kwandong University, Incheon 22711, Korea; dcastle@hanmail.net; 3 Department of Medicine, Catholic Kwandong Graduate School, Gangneung-si 25601, Korea; fbdlxk@naver.com; 4 Department of Orthopedic Surgery, Gangnam Severance Hospital, Yonsei University College of Medicine, Seoul 06276, Korea

**Keywords:** degenerative osteoarthritis, stem cell therapy, knee, cartilage regeneration

## Abstract

Current clinical applications of mesenchymal stem cell therapy for osteoarthritis lack consistency because there are no established criteria for clinical processes. We aimed to systematically organize stem cell treatment methods by reviewing the literature. The treatment methods used in 27 clinical trials were examined and reviewed. The clinical processes were separated into seven categories: cell donor, cell source, cell preparation, delivery methods, lesion preparation, concomitant procedures, and evaluation. Stem cell donors were sub-classified as autologous and allogeneic, and stem cell sources included bone marrow, adipose tissue, peripheral blood, synovium, placenta, and umbilical cord. Mesenchymal stem cells can be prepared by the expansion or isolation process and attached directly to cartilage defects using matrices or injected into joints under arthroscopic observation. The lesion preparation category can be divided into three subcategories: chondroplasty, microfracture, and subchondral drilling. The concomitant procedure category describes adjuvant surgery, such as high tibial osteotomy. Classification codes were assigned for each subcategory to provide a useful and convenient method for organizing documents associated with stem cell treatment. This classification system will help researchers choose more unified treatment methods, which will facilitate the efficient comparison and verification of future clinical outcomes of stem cell therapy for osteoarthritis.

## 1. Introduction

Degenerative osteoarthritis (OA) is a common health concern worldwide. It is a major cause of disability that can negatively affect the physical and mental well-being of the patient [1]. Years of living with OA and disability were exceptionally high, with 836 and 3039 years per 100,000 old men and old women, respectively [2]. As society ages, the prevalence of degenerative OA increases, and the corresponding social costs will be a problem. Similarly, the importance of treatment for middle-aged patients (50–60 years old) has been emphasized by the corresponding average life expectancy exceeding 80 years.

There are various underlying mechanical and biochemical factors that cause OA [3]. Abnormal loading due to obesity, malalignment or instability of the joints, trauma, and excessive use have been considered as risk factors for the development of degenerative OA [4,5]. Abnormal physical forces on articular chondrocytes interrupt their metabolic processes and promote hypertrophy of chondrocytes, which leads to the undesirable production of proteolytic enzymes such as matrix metalloproteinase 13 (MMP13) [6,7]. A pro-inflammatory environment associated with a damaged cartilage, combined with mechanical stress, increases the release of pro-inflammatory cytokines and soluble mediators, thereby accelerating the degradation of the cartilage matrix [5,8]. Due to its progressive nature, degenerative OA eventually develops into the loss of major joint function.

In the initial and moderate stages of degenerative OA, conservative treatments such as exercise, physical therapy, and taking anti-inflammatory or analgesic medicines are frequently suggested, and as the disease progresses, steroid or hyaluronic acid injections have been considered [9,10,11,12]. Several treatment options, such as arthroscopic surgeries, knee osteotomy, and unicompartmental knee arthroplasty, are available for treating the severe stage of the disease [13,14,15]. The final treatment for knee OA is total knee arthroplasty (TKA). However, artificial joints have limited durability, which is controlled by various host factors, and often require revision [16,17]. Recently, cell-based therapies have been used to alleviate and reduce the progression of degenerative OA [18,19,20]. In particular, mesenchymal stem cells (MSCs), which can be differentiated into various types of functional tissue cells, have shown superior ability to regenerate damaged cartilage, as well as to provide significant and clinically relevant pain relief [21,22]. The clinical applications of MSCs, for the treatment of OA of the knee, utilize their ability to differentiate chondrocytes [23].

The therapeutic mode of action of MSCs is recognized in two ways: direct differentiation from MSCs to chondrocytes and optimization of the intra-articular environment [24]. Strategies for enhancing the capacity of chondrogenic differentiation include various growth factors, chemical materials, and scaffold applications [25,26]. Its paracrine effect without direct contact is also drawing attention due to its ability to increase cartilage regeneration [27]. Several meta-analyses of randomized controlled trials have reported that MSC therapy is effective in reducing pain and improving the clinical symptoms of OA [28,29,30].

Despite the therapeutic potential of MSCs, their current clinical applications show a lack of consistency and low homogeneity in terms of detailed treatment methods [20,28]. Various methods of stem cell treatment have been attempted using several combinations of processes, such as cell source selection, cell preparation, cell delivery methods, lesion site preparation, and concomitant treatment. This diversity of methods has made it difficult to integrate and comprehensively compare research results. The reason for this may partly be the lack of standardized criteria for applying clinical processes.

Therefore, the delineation of procedures is necessary to validate vague descriptions and irregular combinations of MSC treatment methods. The purpose of this study was to organize and categorize scattered MSC treatment methods by reviewing clinical trials employing MSC treatment for degenerative OA of the knee. The aim of this categorization is not to investigate the superiority among different methods but to organize heterogeneous and non-systematic trials to help researchers find more effective and appropriate methods for MSC therapy.

## 2. Search and Selection of Clinical Trials Applying Stem Cell Treatment

**Figure 1:**
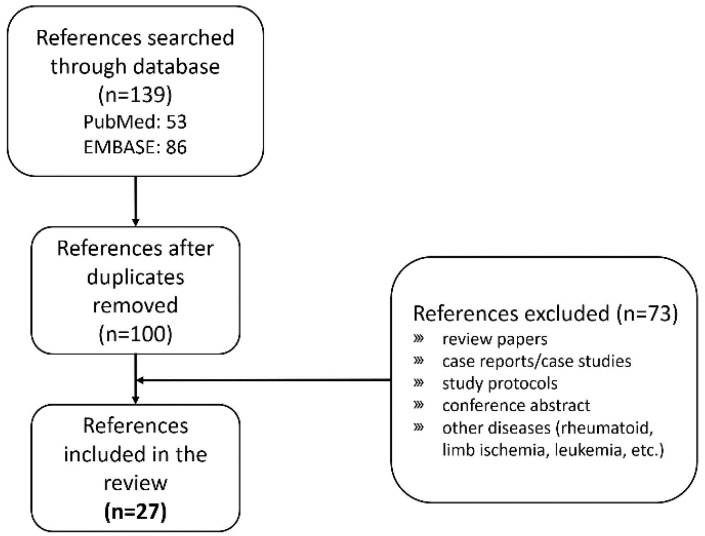
Flow chart entailing the literature search process.

We selected research papers on clinical trials that treated degenerative OA of the knee with MSCs and reviewed the treatment process to categorize the method. The PubMed and EMBASE databases were searched to collect and retrieve relevant research papers (Figure 1). The search keywords included combinations of “osteoarthritis”, “cartilage defect”, “knee”, “stem cells”, “mesenchymal stem cell”, “vascular stromal fractions”, “randomized controlled trials”, and “double-blinded”. There were 100 papers searched based on these combinations of keywords, and 27 research papers were selected after a detailed review. The exclusion criteria are shown in Figure 1.

## 3. Method Categorization of Current Stem Cell Treatment Methods for Degenerative OA

**Figure 2:**
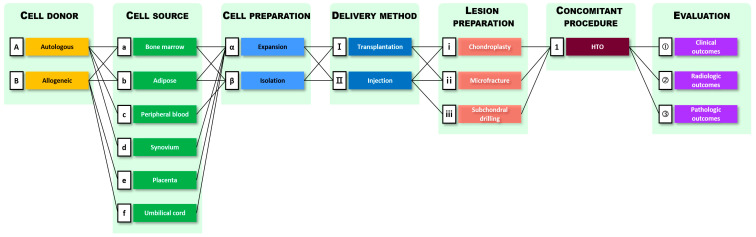
Categorization of MSC therapy procedures based on the treatment methods used in studies. There are seven main categories, and each category is further divided into subcategories. The letter, symbol, or number mentioned on the left of each subcategory represents the classification code.

Based on the treatment methods shown in the chosen studies, the MSC therapy procedures were classified into seven main categories and further subcategories (Figure 2). In Figure 2, combinations of subcategories observed in the 27 clinical trials are indicated by connected lines.

As shown in Figure 2, MSC therapy procedures can be classified into seven categories: selection of cell donor and cell source, cell preparation, delivery method, lesion preparation, concomitant procedure, and evaluation. Cell donors have been sub-categorized as autologous and allogeneic, and cell sources mainly used in these studies were the bone marrow, adipose tissue, peripheral blood, synovium or infrapatellar fat pad, placenta, and umbilical cord (tissue or blood). Cell preparation can be divided into two subcategories: expansion, which requires an incubation process, and isolation accompanied by simple centrifugation or filtration. The delivery methods can be divided into transplantation and injection. Transplantation includes attachment of MSCs to cartilage defects using matrices such as scaffolds or collagen sheets, whereas injection corresponds to indirect transplantation of MSCs into joints under arthroscopic observation. The lesion preparation category describes how to prepare a defective cartilage area where MSCs are to be transplanted. Chondroplasty refers to the trimming and removal of unstable flaps from defective cartilage. Microfracture and subchondral drilling are performed to stimulate the subchondral region to increase its innate ability to heal by releasing bone marrow and circulating blood. The concomitant procedure category describes adjuvant surgery, such as a high tibial osteotomy (HTO), used to correct the malalignment of joints. The evaluation process included clinical, radiological, and pathological outcomes.

**Figure 3:**
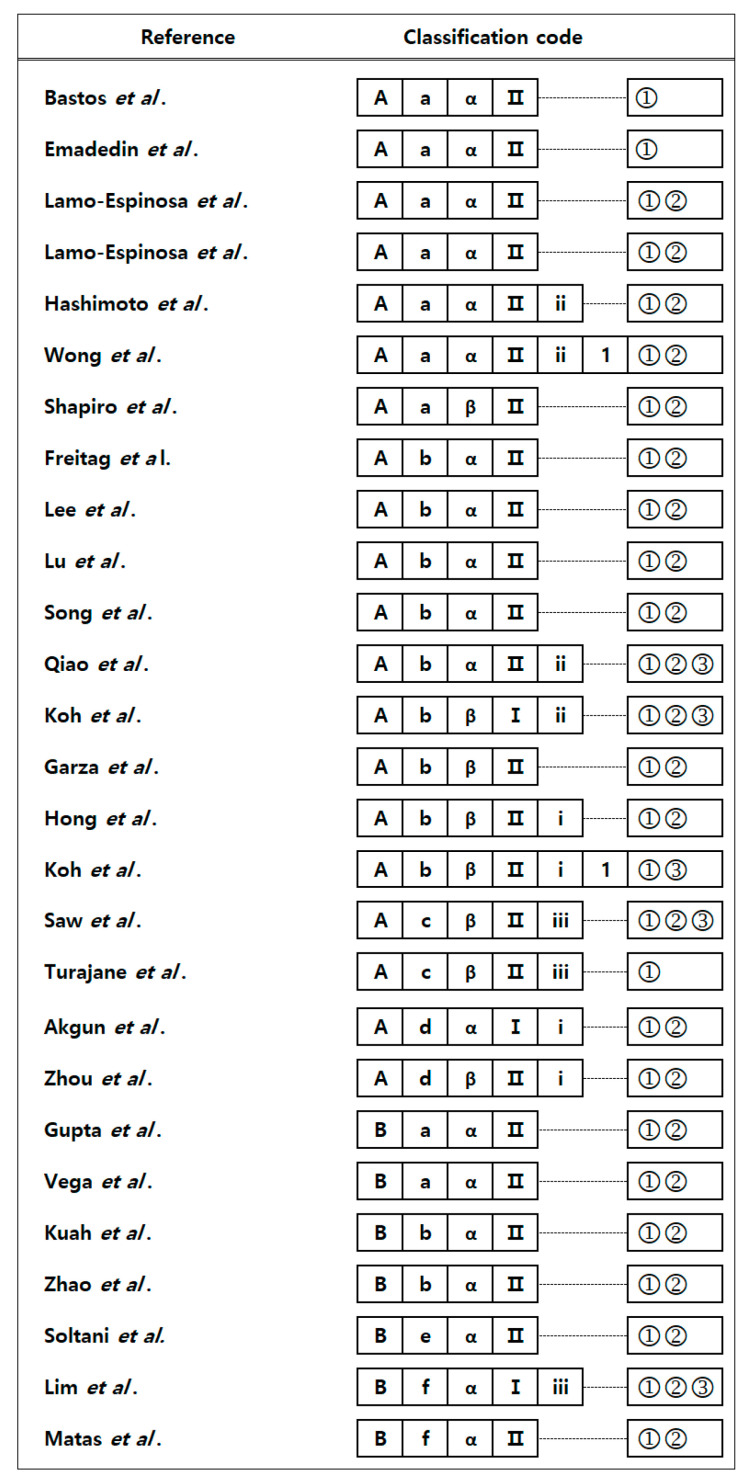
Classification codes of the references reviewed in this paper. A, autologous; B, allogeneic; a, bone marrow; b, adipose; c, peripheral blood; d, synovium; e, placenta; f, umbilical cord; α, expansion; β, isolation; I, transplantation; II, injection; i, chondroplasty; ii, microfracture; iii, subchondral drilling; 1, HTO; ①, clinical outcomes; ②, radiologic outcomes; ③, pathologic outcomes.

Figure 3 shows the classification codes given to the 27 clinical trials reviewed in this study. Classification codes were assigned to provide a useful and convenient method for organizing reports on MSC treatment. For example, if a study involved the injection of isolated autologous bone marrow stem cells into knee cartilage lesions after microfracture followed by evaluation with magnetic resonance imaging (MRI), the classification codes of the corresponding document are A-a-β-II-ii-②. In this way, references can be assigned by a combination of classification codes to make it easier to compare and evaluate clinical findings within the same category.

**Figure 4:**
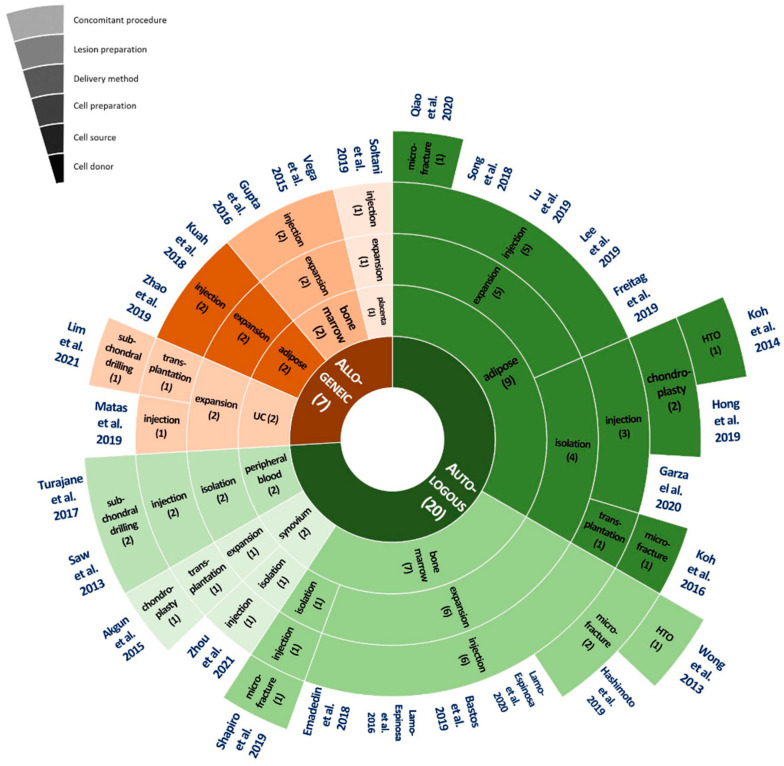
Distribution of treatment methods in clinical trials reviewed in this paper. Values in parenthesis represent the number of clinical trials employing the corresponding treatment method. HTO, high tibial osteotomy; UC, umbilical cord.

The distribution of the current MSC treatment methods observed in 27 clinical trials is presented in Figure 4. Figure 4 does not include the distribution of evaluation methods because all clinical trials adopted similar evaluation methods (Figure 3). Each of the six layers of the circle corresponds to one of six main categories, and each layer is divided into subcategories based on the number of corresponding studies. Based on the distribution circles, we can see how many trials employed the corresponding treatment methods in each subcategory. References corresponding to the combinations of each subcategory are shown at the top of the layers.

## 4. Treatment Methods by Process

### 4.1. Patient Selection and Randomization

The sample sizes of the intervention and control groups were determined either by considering the feasibility of the procedures or, preferably, by formal statistical power calculations. Statistical sample size calculations have been estimated to detect an effect size of 0.6, with a power of 80–85% and a type I error probability of 5% [10,31,32,33,34,35]. Table 1 lists the inclusion and exclusion criteria for the trials for patient selection. Depending on the purpose and method of the study, the inclusion and exclusion criteria can be modified.

In many of the trials reviewed in this study, the patients’ ages ranged from 16 to 80 years. Age is one of the most important risk factors for OA progression. OA progresses to the end-stage at a rate of 1.2% in 45–55-year-old patients, whereas this rate increases to 5.1% in those over 75 years [36]. In addition, the highest incidence rates for degenerative OA have been observed in patients aged 55–65 years [37]. Therefore, it would be desirable to narrow this range and enroll patients in the age group of 45–65 years. The gender distribution of clinical trials has shown that in many cases, the number of female subjects is higher, presumably due to the higher prevalence of OA in women [2]. There are studies that show racial and ethnic differences in pain, function, and radiographic features in older adults with knee OA [38,39]. Therefore, researchers should consider presenting the racial/ethnic background in their patient demographics and analyzing it as the factors that may influence the results.

In clinical trials, it is common to limit the affected joint to a single knee, but it is also possible to assess the other joints simultaneously. To assess the therapeutic effects associated with other knee joints, appropriate measurement methods should be included. Patients with knee malalignment or deformities were excluded unless there were concomitant surgical procedures to reduce mechanical factors. The progression of OA can be significantly affected by comorbidities in patients. For adults with OA, it has been estimated that 31% have five or more chronic conditions, with the most common being cardiovascular disease, diabetes mellitus, and hypertension [36]. It is known that those who rate their joint function as worse are most likely to be affected by comorbid chronic conditions [36]. The significance of the effect of interaction between OA and comorbidities should be taken seriously, and patients with comorbidities should be carefully examined and excluded. A list of comorbidities is presented in Table 1.

To implement randomized clinical trials, patients selected for the study should be randomly assigned to the treatment and control groups. For randomization, most of the randomized clinical trials reviewed in this paper used simple or block randomization using commercially available tools such as CapTool^®^ randomization service (Mebix Cooperation, Tokyo, Japan), Excel software (Microsoft, Washington, DC, USA), a web-based automated random number generator (www.randomizer.org, accessed on 1 October 2021), PROC PLAN in SAS software (SAS Statistical Institute, Cary, NC, USA), and SPSS 20.0 (IBM Corporation, NY, USA). Several studies simply made patients choose one of the two identical envelopes with an assignment to the treatment or control group. This randomization process is essential for the study outcomes to provide a high level of evidence.

### 4.2. Cell Donor

#### 4.2.1. Autologous MSCs

Autologous MSCs can be the best treatment option because they can minimize the risk of immune rejection [40]. According to our investigation, 20 out of 27 clinical trials used autologous MSCs for MSC therapy (Figure 4). The most common autologous MSC sources are adipose tissues and the bone marrow. Autologous MSCs are isolated or expanded under good manufacturing practice conditions [33]. The expansion process increases the number of cells by cultivation, and the entire process takes 5 to 15 days to reach the desired cell concentrations. Cell expansion of MSCs was performed in 12 out of 20 clinical trials using autologous MSCs. Using autologous MSCs may have the limitation of difficulty in keeping patients blinded during their allocation to the intervention and control groups [35]. Some trials conducted the extraction of autologous MSCs from patients, in a control group, who did not receive MSC treatment; however, from an ethical standpoint, it would be desirable to find a way to minimize the application of this unnecessary process.

For the use of homologous MSCs for clinical trials, researchers should be aware of the regulation guidelines of each country. In some countries, the use of homologous MSCs can be strictly regulated. In South Korea and the United States, homologous MSCs should be minimally manipulated, without the use of cell culture or by adding other materials. In the United States, cell-based investigational products must go through the FDA review process for safety and effectiveness in the context of well-controlled human studies [41]. On the other hand, the European Union recognizes stem cell therapy as “advanced treatment of medical product”, not “treatment”, and allows hospital exemptions so that individual patients can receive stem cell treatment [42].

#### 4.2.2. Allogeneic MSCs

There are several sources of allogeneic MSCs. Adipose tissue-derived or bone marrow-derived allogeneic MSCs can often be obtained from other patients participating in the trial [43]. Allogeneic MSCs from the placenta or umbilical cord can be donated by a full-term healthy mother, with consent. In addition, several medicinal products are available as allogeneic MSC sources. CARTISTEM^®^ (MEDIPOST, Co., Ltd., Seongnam-si, Korea), constituting allogeneic umbilical cord blood-derived MSCs, was approved in South Korea in 2012 and has completed the US Food and Drug Administration phase 1/2a clinical trials [44]. Progenza (Regeneus Ltd., Sydney, Australia), which completed a phase 1 trial in Australia, was made from expanded allogeneic MSCs from human adipose tissue and contains the bioactive secretions of the cells [45]. Stempeucel^®^ (Stempeutics Research, Bangalore, India), consisting of human bone marrow-derived adult allogeneic MSCs, has been tested in several phase 3 clinical trials and classified as an advanced therapy medicinal product in the European Union [46]. In the United States, the only stem cell-based products that are FDA-approved for use consist of blood-forming stem cells (hematopoietic progenitor cells) derived from cord blood; such products are intended for limited use in patients with disorders that affect the hematopoietic system [40].

The application of allogeneic MSCs can be more convenient than that of autologous MSCs because the process does not require invasive MSC collection and saves the time associated with waiting for cell expansion [43]. A possible limitation in implanting allogeneic MSCs is host immune rejection; however, MSCs can be tolerated because of their immunomodulatory characteristics [43].

### 4.3. MSC Sources and Preparation

#### 4.3.1. Bone Marrow-Derived MSCs

The bone marrow is the gold standard for deriving MSCs for transplantation [47]. It is a reliable source of MSCs, and such MSCs have superior osteogenic power [32,48,49,50]. In the clinical trials reviewed in this study, 7 [32,33,48,49,50,51,52] out of 20 trials with autologous MSC treatment and 2 [43,46] out of 7 trials using allogeneic MSC treatments utilized bone marrow as the MSC source (Figure 4).

To qualify and define the number and characteristics of the derived MSCs, it is recommended to follow the minimal criteria to define human MSCs, proposed by the International Society for Cellular Therapy. MSCs must be plastic-adherent in standard culture conditions and must express CD105, CD73, and CD90, as well as lack the expression of CD45, CD34, CD14, CD11b, CD79a, CD19, and human leukocyte antigen-isotype DR surface molecules [46]. Differentiation of MSCs into osteoblasts, adipocytes, and chondroblasts should be confirmed in vitro. Although these criteria will probably require modifications, these minimum criteria will promote a more uniform characterization of MSCs and facilitate the exchange of data among investigators [53].

**Figure 5:**
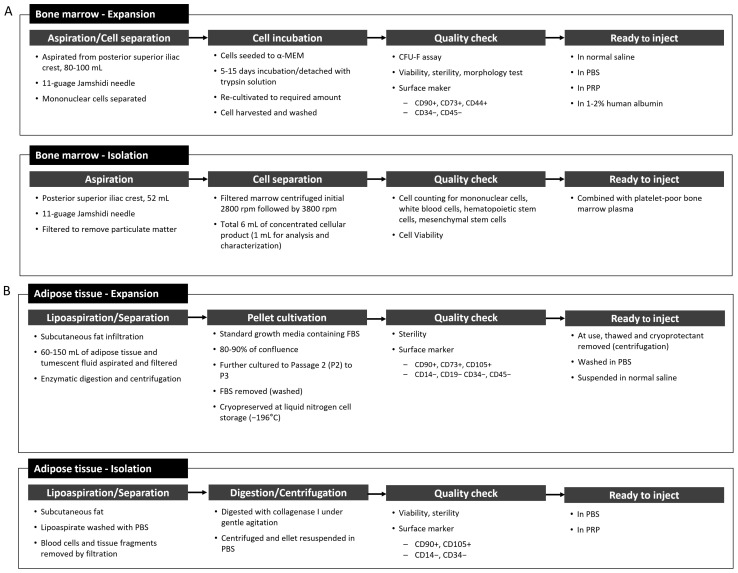
MSC preparation process in the clinical trials for (**A**) bone marrow and (**B**) adipose tissue. α-MEM, Alpha Modification of Eagle’s Minimum Essential Media; CFU-F assay, colony-forming unit fibroblast assay; PBS, phosphate-buffered saline; PRP, platelet-rich plasma; FBS, fetal bovine serum.

The bone marrow was collected from the iliac crest using an 11G Jamshidi needle under spinal nerve block and sedation. In the aspirated samples, 0.01% of the 6 × 10^6^/mL nucleated cells were found to be bone marrow MSCs [54]. In the majority of studies, the bone marrow was expanded to reach the desired concentration. The general expansion and isolation processes reported in clinical trials are shown in Figure 5A.

#### 4.3.2. Adipose Tissue Derived MSCs

Adipose tissue has been recognized as a potential source of autologous MSCs because of the relative ease of harvest, the abundance of MSCs, and high chondrogenic potential in comparison to other sources, such as the bone marrow [31]. Adipose-derived MSCs tend to proliferate more rapidly, are less susceptible to senescence [40,54], and are not affected by the patient’s age, sex, and physiological status [55]. A yield of approximately 5% MSCs can be expected in 2 × 10^6^ nucleated cells isolated from 1 g of adipose tissue, which is significantly higher than that isolated from other tissues [56]. Adipose tissue is retrieved mostly by lipoaspiration from the abdominal subcutaneous fat. In the clinical trials reviewed, 9 [21,31,34,35,40,57,58,59,60] out of 20 trials with autologous MSC treatment and 2 [45,61] out of 7 trials with allogeneic MSC treatment used adipose tissue (Figure 4). The general preparation procedures for adipose-derived MSCs reported in clinical trials are shown in Figure 5B.

#### 4.3.3. Peripheral Blood Stem Cells and Synovium-Derived MSCs

In normal peripheral blood, less than 0.1% of hematopoietic stem cells exist. Therefore, the production and release of functional neutrophils from the bone marrow into the bloodstream is stimulated by the daily administration of human granulocyte colony-stimulating factor for several days before peripheral blood collection. It has also been found that there is an abundant and feasible mixture of MSCs and endothelial progenitor cells in autologous activated peripheral blood stem cells [62]; therefore, peripheral blood stem cells are included in the cell source category in this study. Peripheral blood stem cells are collected via apheresis by central venous access, and mononuclear white blood cells and peripheral blood stem cells are separated. The data showed that 0.29% of 10^3^ total nucleated cells/mL were CD34+ and CD105+ stem cells [62]. As there has been a shift in clinical practice from the use of bone marrow to peripheral blood as the donor for hematopoietic stem cells over the last decade [63], the use of PBSCs to treat OA is expected to increase.

**Figure 6:**
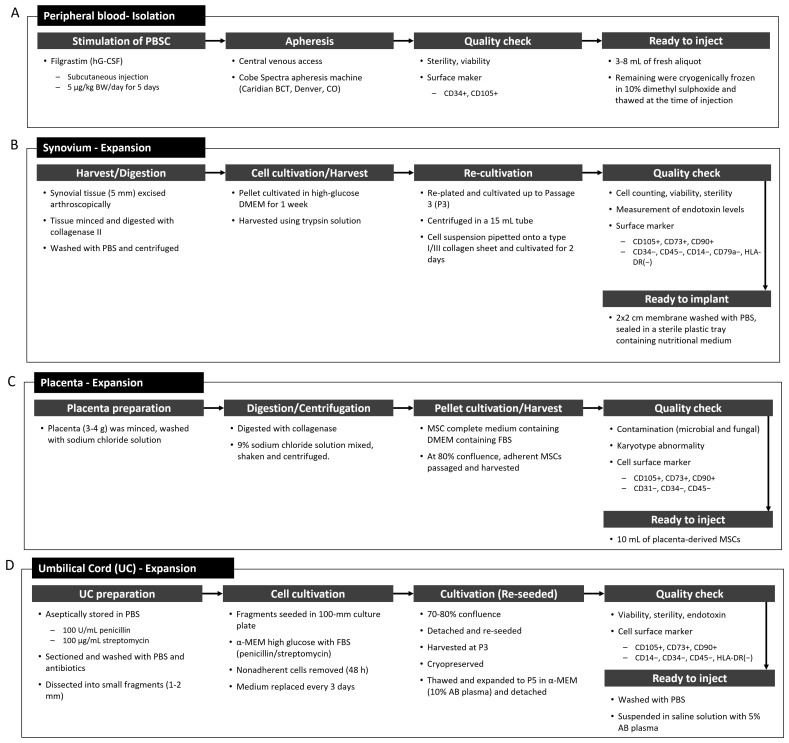
Procedures listed in the clinical trials for the preparation of MSCs from the (**A**) peripheral blood, (**B**) synovium, (**C**) placenta, and (**D**) umbilical cord.

Synovium-derived MSCs have also been considered to have potential applicability for cartilage regeneration, owing to their superiority in chondrogenesis and osteogenesis [64,65]. It has been reported that the gene profiles of MSCs from intra-articular tissues are closer to chondrocytes than those from extra-articular tissues [66]. Synovium-derived MSCs can be extracted from the suprapatellar pouch, infrapatellar fat pad, and medial outer and medial inner regions [64,67,68]. General preparation procedures for PBSCs and synovium-derived stem cells in clinical trials are shown in Figure 6A,B, respectively.

#### 4.3.4. Placenta- and Umbilical Cord-Derived MSCs

Placenta- and umbilical cord-derived MSCs are the major sources of allogeneic MSCs, and they have become more accessible sources [69,70,71]. Umbilical cord-derived MSCs have demonstrated higher clonogenicity, proliferation, and migration potential than bone marrow-derived MSCs, as well as improved secretion of relevant chondrogenic factors [70]. In addition, umbilical cord-derived MSCs have shown high expansion capacity, which provides enough cells for therapeutic applications [44]. The placenta or umbilical cord can be obtained from a full-term healthy mother, with consent. General preparation procedures used in the clinical trials for the isolation of MSCs from the placenta and umbilical cord are shown in Figure 6C,D, respectively.

### 4.4. Delivery Methods for MSCs

#### 4.4.1. Transplantation

Transplantation is a process in which isolated or cultured MSCs on a supporting material, such as collagen sheets or scaffolds, are directly placed and fixed in the lesion area of the cartilage. This method minimizes the dissipation of MSCs in the graft so that they can differentiate into chondrocytes in cartilage. Among the trials reviewed, only two trials using autologous MSCs employed the transplantation method (Figure 4). Koh et al. (2016) used a commercially available fibrin glue product (Greenplast; Green Cross, Yongin, Korea) containing lyophilized human plasma fibrinogen and thrombin solution loaded with MSC suspension [21]. When the two solutions were mixed, the glue instantly formed a gel, and the gel was implanted onto the cartilage lesion surface under arthroscopic guidance [21]. Akgun et al. (2015) cultivated MSCs on the surface of type I/III collagen membranes (2 × 2 cm, Chondro-Gide; Geitschlich Biomaterials, Wolhusen, Switzerland), then directly transplanted the membrane to the lesion area on the subchondral bone, and fixed cells using fibrin sealant [67].

Recently, the paracrine effects of transplanted MSCs have attracted more attention than the differentiation of MSCs into chondrocytes. Donor MSCs were not maintained after one year in the host tissue of patients who had received MSC injections for different diseases, and other studies reported a consistent lack of engraftment of transplanted MSCs in the host tissues [8]. The lack of engraftment may be partly due to the deficiency of transplantation technology and its infrequent use compared to injections.

#### 4.4.2. Injection

Injection of MSCs is the most common method to indirectly deliver MSCs into the intra-articular space. The injection is generally performed using a 19-gauge needle (under local anesthesia) under arthroscopic guidance. Injected MSCs are expected to be localized in cartilage lesions to regenerate cartilage by differentiation. More importantly, injected MSCs exhibit a paracrine effect, which establishes a regenerative environment by secreting various chemokines and cytokines, which are the influencing factors.

Table 2 illustrates the types of supplements transplanted with MSCs. Most substances, such as hyaluronic acid, platelet-rich plasma, and saline, have been known to improve the symptoms of OA by themselves; therefore, it is important to exclude their background effects and carefully analyze the clinical outcomes. The injection of MSCs can include single or multiple doses of different concentrations; however, the therapeutic effects of treatment with different doses and concentrations are controversial.

### 4.5. Lesion Preparation

Lesion preparation is the process of producing stable cartilage lesion areas and stimulating the bone marrow of the subchondral bone, where MSCs are transplanted. Subchondral bone plays a key role in providing the deepest layers of articular cartilage with nutrient supply and removal of waste products [72]. The widely used preparation methods include chondroplasty, microfracture, and subchondral drilling. Chondroplasty removes all damaged and unstable cartilages, such as articular cartilage fragments, chondral flaps, or osteophytes, to form stable cartilage [35,58]. Chondroplasty can be performed alone to make the subchondral region uniform and visible. This process is accompanied by microfractures and subchondral drilling. Microfractures are performed by penetrating holes 3–4 mm apart, deep enough to develop the “fat-pearls” of the subchondral bone [34]. Subchondral drilling is processed by multiple drillings of 2.00 mm diameter at a depth of 4–6 mm after meticulous removal of the calcified cartilage layers of the subchondral bone and formation of a stable edge of good cartilage [62]. Multiple drilling techniques use a high speed of approximately 10,000–400,000 rpm [73], which can cause damage to the surrounding tissue due to heat generation and frictional forces. Microfracture and subchondral drilling are widely used bone marrow stimulation techniques for articular cartilage repair in clinical circumstances, and they have therapeutic effects in treating OA. The damaged environment releases signals to recruit dormant pericytes to the sites, and the endogenous progenitor secretes the profile of chemical factors to create a regenerative environment [8].

### 4.6. Concomitant Procedure

Concomitant surgical procedures are necessary for patients with OA due to mechanical factors. Surgical procedures that can be accompanied by MSC therapy include HTO. HTO is a surgical procedure used to realign the weight-bearing line in the coronal plane for patients with internal deformity due to OA. Meniscectomy and anterior cruciate ligament reconstruction can possibly be included under the concomitant procedure category, although these processes were not observed in the clinical trials reviewed. A concomitant procedure is necessary if mechanical factors are the underlying cause of OA because MSC treatment alone cannot improve the symptoms under these circumstances. Since the surgical procedure to remove mechanical factors causing OA is remarkably effective, the therapeutic contribution of MSC treatment is relatively underestimated. What is certain is that the two procedures have their roles, which are the removal of the factors causing mechanical OA and the promotion of cartilage regeneration.

### 4.7. Evaluation Process

**Figure 7:**
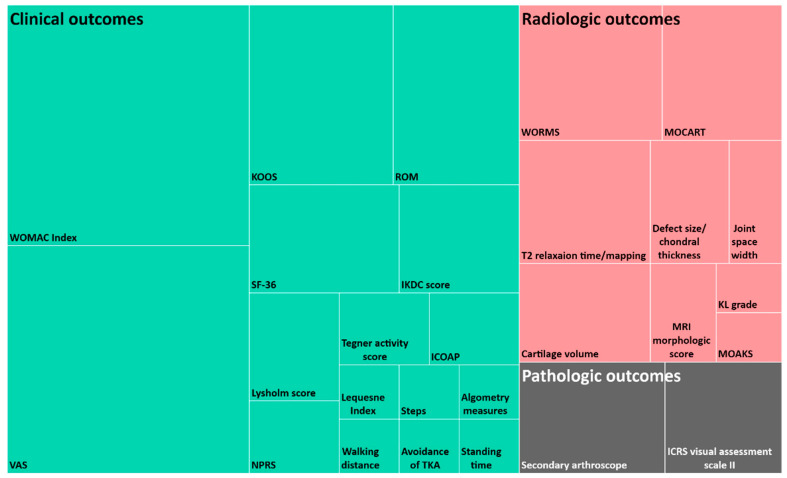
Frequency of evaluation methods under each outcome category.

To evaluate the safety and efficacy of MSC treatment, all patients were followed up for a minimum of 12 months in the clinical trials reviewed in this study. The efficacy of clinical trials for OA can be evaluated by examining clinical outcomes, radiological outcomes, and pathological outcomes. These outcome assessments were conducted to determine changes in the pain, function, and structure of the joint before and after treatment. It has been shown that most clinical trials reviewed in this study employed clinical and radiologic outcome evaluations. The various measurement methods used for each evaluation subcategory and the frequency of each method under the three subcategories are shown in Figure 7.

Pain in the joints is mainly measured using a visual analog scale (VAS). It can assess pain during the activity and rest periods separately. Since the pain and function of joints are interdependent and cannot be separated in principle, it is recommended to use a tool that can measure two factors simultaneously. The Lequesne Index and Western Ontario and McMaster Universities (WOMAC) score can be utilized as self-survey assessment tools for evaluating joint pain, function, and stiffness. The range of motion (ROM) and knee injury and OA outcome score (KOOS) are also useful tools to evaluate the improvement of symptoms and functions.

Structural improvements can be more directly assessed through radiologic observations using X-ray and MRI scans. In addition to MRI imaging of the knee joint, an MRI morphologic scoring system specified by criteria, findings, and score at the same time can be utilized for a better quantitative evaluation of the results. Since a significant part of the evaluation process for clinical outcomes is empirical and patient driven, attempts are being made to present the results of treatment more quantitatively. The measurement of T2 relaxation time using MRI is a way to quantify the condition of the joint cartilage. The T2 mapping system measures the difference in water mobility in the collagen framework and expresses red to yellow color for poor cartilage and blue color for good cartilage [61]. Vega et al. (2016) divided the femoral and tibial condyles into eight sections, measured the monitored T2 relaxation time, and attempted to quantify the effects before and after MSC treatment [43]. Zhao et al. (2019) also analyzed various compositional indexes such as glycosaminoglycan, extracellular matrix, T2 mapping, T1rho mapping, diffusion-weighted imaging, and apparent diffuse coefficient before and after MSC treatment [61].

Secondary arthroscopy and histologic analysis in the evaluation of pathologic outcomes are methods that directly observe cartilage regeneration. For histologic analysis, histologic scores can be calculated using the organizational score system (International Cartilage Research Society Visual Assessment Scale II score). The use of secondary arthroscopy and histologic analysis through biopsies should be minimized, and appropriate patient consent should be obtained because of the ethical reasons related to the invasive nature of these processes.

The representative outcomes of the clinical trials reviewed in this study are presented in Table 3. As shown in Table 3, most of the studies reported clinically effective pain relief and symptom improvement. In terms of regeneration of cartilage, several studies showed an increase in the volume and thickness of cartilage, and studies that could not observe this increase showed that cartilage damage did not progress compared to controls that did not undergo stem cell therapy.

The types of clinical outcomes and the way of presenting the results of each study have a great deal of diversity. Regarding future study design, researchers should consider presenting clinical outcomes in a more uniform manner. It is suggested to include the top two most frequently used clinical outcomes, VAS and WOMAC as illustrated in Figure 7, and at least one of the radiological outcomes to prove the presence of cartilage regeneration.

## 5. Conclusions

The purpose of this study was to increase the consistency of future MSC therapies for OA by categorizing the current treatment methods.

The treatment methods can be divided into seven categories: cell donor, cell source, cell preparation, delivery methods, lesion preparation, concomitant procedures, and evaluation. Based on these procedures, classification codes were assigned to each subcategory. Stem cell donors were subdivided into autologous and allogeneic, and stem cell sources included bone marrow, adipose tissue, peripheral blood, synovium, placenta, and umbilical cord. MSCs were prepared through cell expansion or isolation processes. They were attached to cartilage defects using matrices or injected into the joints under arthroscopic observation. The lesion preparation category was divided into three subcategories: chondroplasty, microfracture, and subchondral drilling. The concomitant procedure category describes adjuvant surgery, such as high tibial osteotomy.

Additional parameters can be added in future clinical studies. Cell sources can include MSCs from molar cells, amniotic fluid, and induced pluripotent stem cells. Some alternative cell sources, such articular cartilage progenitors and chondrogenic progenitor cells that have the potential to treat OA can also be considered as new parameters [74,75]. The addition of various treatment factors, such as PRP, hyaluronic acid, and some growth factors, can be considered as a new category. Low-intensity pulsed ultrasound (LIPUS), meniscectomy, and anterior cruciate ligament (ACL) reconstruction can be attempted as additional concomitant procedures. Since current MSC therapies are inconsistent and lack homogeneity, the classification system proposed in this study is expected to facilitate the efficient comparison and verification of clinical outcomes from MSC therapy for degenerative OA. Furthermore, if the analysis of the clinical results for each category is accumulated, the optimal combinations of efficient stem cell treatment methods can be found.

## Figures and Tables

**Table 1:** Inclusion and exclusion criteria for the selection of patients in randomized clinical trials.

Inclusion Criteria	
General	Males and females aged 18–75 years
	Patients in stable health
OA diagnosis	Symptomatic and radiographic OA
	Kellgren–Lawrence grade 2 to 4
	ICRS articular injury classification ≥ 3
Exclusion criteria	
General	BMI ≥ 40 kg/m^2^
	Pregnancy or lactation
	Mental disorder
	Those participating in another clinical trial
	Chronic treatment with immunosuppressive or anticoagulant drugs
	Alcoholism, drug abuse
	Unable to answer subjective questionnaires and inability to provide informed consent
OA diagnosis	Secondary arthritis (related to rheumatoid arthritis, spondyloarthritis, or previous major knee traumas)
	Mechanical pain caused by meniscal tears (including flap tears, bucket-handle tears, and complex tears)
	Bi-compartmental and tri-compartmental OA
Malalignment/ deformities	Malalignment of the knee from femoral causes
	Fixed flexion deformity of the knee
	Collateral ligament instability
	Joint line congruity angle of more than 2°
	Severe mechanistic extra-articular deformation (varus/valgus, >15°)
Treatment related	Allergic reaction to components of study treatment and/or study implantation procedure
	Unable to tolerate magnetic resonance imaging scans
Previous treatment	Previous meniscectomy/significant partial meniscectomy
	Prior stem cell treatment
	Intra-articular injection of hyaluronic acid or corticosteroid in the preceding 2 months
	Undergone previous cartilage procedures, such as microfracture or chondroplasty
	Arthroscopy or intraarticular infiltration in the last 6 months
	Corticosteroid treatment in the 3 last months
	Nonsteroidal anti-inflammatory drug therapy in the last 15 days
	Previous surgical treatment for anterior and/or posterior cruciate ligament reconstruction within 2 months
Other diseases/ comorbidities	History of autoimmune disease
	Malignancy, organ failure
	Cardiovascular disease, hypertension
	Positive viral markers (HIV, HBV, HCV, and HTLV-1/2), syphilis
	Bleeding disorder, i.e., hemophilia
	Poorly controlled diabetes mellitus

ICRS, International Cartilage Repair Society; BMI, body mass index; HIV, human immunodeficiency virus; HBV, hepatitis B virus; HCV, hepatitis C virus; HTLV, human T-cell lymphotropic virus type; OA, osteoarthritis.

**Table 2:** Substances implanted or injected with mesenchymal stem cells.

Delivery Method	Substances	
Transplantation	Collagen matrix	Collagen type I/III membrane
		Collagen sheet
	Fibrin glue	Fibrin glue product (fibrinogen and thrombin)
Injection	Basal medium	Minimum essential medium
		Normal saline
		Human serum
		Albumin
		Platelet poor plasma
	Hyaluronic acid	Hyalone^®^ (Hyaluronic acid sodium salt 4 mL/60 mg)
		Artz^®^ (Hyaluronic acid sodium salt)
	Platelet-rich plasma	Concentrated platelets from the autologous blood
	Growth factor	Human granulocyte colony-stimulating factor

**Table 3:** Representative outcomes of clinical trials reviewed in this study.

References	Patients	Follow Up	MSC Doner and Source	Number of MSCs	Delivery Method	Lesion Preparation/Concomitant Procedure	Clinical, Radiological, and Histological Outcomes	Conclusions
Bastos et al. (2019)	*n* = 47	12 months	Autologous bone marrow	4 × 10^6^	Injection	None	- Improved global KOOS scores - No significant differences in ROM	Treatments were effective in improving the function and decreasing symptoms.
Emadedin et al. (2018)	*n* = 43	6 months	Autologous bone marrow	4 × 10^7^	Injection	None	- Improved VAS and WOMAC total scores - Increased painless walking distances - Increased degree of knee flexion	Significant and clinically relevant pain relief was observed.
Lamo-Espinosa et al. (2016)	*n* = 30	12 months	Autologous bone marrow	1 × 10^7^ 1 × 10^8^	Injection	None	- Improved VAS and WOMAC total scores - Increased knee ROM for flexion and extension - Not decreased knee joint space - Improved WORMS only at low dose treatment	Clinical and functional improvement of knee OA was observed.
Lamo-Espinosa et al. (2020)	*n* = 56	12 months	Autologous bone marrow	100 × 10^6^	Injection	None	- Improved VAS and WOMAC scores - No significant changes in X-ray scan and WORMS	BM-MSC injection with PRP was a viable therapeutic option in the treatment of OA of the knee.
Hashimoto et al. (2019)	*n* = 11	48 weeks	Autologous bone marrow	10 × 10^6^ 100 × 10^6^	Injection	Microfracture	- No significant difference in IKDC scores - Improved KOOS QOL scores - No difference in T2 values with MSC doses - Improved MOCART scores	A better quality of articular surface and improved symptomatic cartilage defect of the knee was observed. High dose (100 × 10^6^) was more effective.
Wong et al. (2013)	*n* = 56	24 months	Autologous bone marrow	1.46 × 10^7^	Injection	Microfracture/HTO	- Improved IKDC, Lysholm, and Tegner scores - Improved MOCART scores	The treatment was effective in improving both short-term clinical and MOCART outcomes.
Shapiro et al. (2018)	*n* = 25	12 months	Autologous bone marrow	1.7 × 10^5^ (MSCs) 2.2 × 10^7^ (HSCs)	Injection	None	- Not improved ICOAP total pain and VAS pain scores - Not improved medial joint line measurement - Not improved MRI T2 values	BMAC is safe to perform but showed no superiority to saline injection. MRI cartilage sequences failed to show regenerative benefit.
Freitag et al. (2019)	*n* = 30	12 months	Autologous adipose	1 ×10^8^ 1 × 10^8^ × 2	Injection	None	- Improved NPRS, KOOS (pain), and WOMAC scores - Improve MOAKS only at single-injection group	Clinically significant pain and function improvement was observed. MOAKS indicated that the disease progression was modified.
Lee et al. (2019)	*n* = 32	6 months	Autologous adipose	1 × 10^8^	Injection	None	- Improved VAS pain, WOMAC scores - No significant change of the size of cartilage defects - No significant changes in K-L grade, joint space width, and HKA angle	Satisfactory functional improvement and pain relief was observed. The treatment inhibited the progression of cartilage defects.
Lu et al. (2019)	*n* = 47	13 months	Autologous adipose	5 × 10^7^ × 2	Injection	None	- Improved VAS, WOMAC, and SF-36 scores - Increased total volume of articular cartilage	Treatments proved significant improvements in joint function, pain, quality of life, and cartilage regeneration.
Song et al. (2018)	*n* = 14	96 weeks	Autologous adipose	1 × 10^7^ × 3 2 × 10^7^ × 3 5 × 10^7^ × 3	Injection	None	- Improved overall WOMAC, mean NRS-11, and SF-36 scores - Increased overall cartilage volume at 72nd weeks.	Treatments was effective in pain reduction, function improvements and the cartilage volume increase. The dosage of 5 × 10^7^ showed the highest improvement.
Qiao et al. (2020)	*n* = 23	24 months	Autologous adipose	5 × 10^7^ × 2	Injection	Microfracture	- Improved WOMAC total score and physical component score of SF-36 - Decreased arthroscopic defect size and MRI defect size at 6 months - Increased cartilage volume and thickness - Increased ICRS II histologic score at 6 months	Function of the knee joint was clinically improved. The treatment promoted the decrease of cartilage defect and cartilage regeneration.
Koh et al. (2016)	*n* = 80	24 months	Autologous adipose	4.97 × 10^6^	Transplantation	Microfracture	- Improved VAS, KOOS pain and symptom subscores - Not improved other KOOS subscores - Improved MOCART tissue scores - Improved ICRS II Histologic scores	Pain and symptom improvements were observed. The appearance of cartilage lesions was improved.
Garza et al. (2020)	*n* = 39	12 months	Autologous adipose (SVF)	1.5 × 10^7^ SVF 3.0 × 10^7^ SVF	Injection	None	- Improved WOMAC total score - No change in cartilage thickness	The treatment significantly decreased knee OA pain and symptoms, and the high dose group showed better results.
Hong et al. (2018)	*n* = 16	12 months	Autologous adipose (SVF)	2.98 × 10^7^	Injection	Chondroplasty	- Improved VAS, WOMAC pain, and ROM - Improved WORMS and MOCART scores	The treatment effectively relieved pain, improved function, and repaired cartilage defects.
Koh et al. (2014)	*n* = 44	24.4 months	Autologous adipose	4.11 × 10^6^	Injection	Chondroplasty/HTO	- Improved VAS, KOOS, and Lysholm scores - Better defect coverage on second-look arthroscopy	Treatments was clinically effective and mildly improved cartilage healing.
Saw et al. (2013)	*n* = 49	18 months	Autologous peripheral blood	2 × 10^7^ × 8 (CD105^+^) 3 × 10^6^ × 8 (CD34^+^)	Injection	Subchondral drilling	- Improved IKDC scores - Improved MRI scores - Increased ICRS II histologic scores	The quality of articular cartilage repair was improved.
Turajane et al. (2017)	*n* = 60	12 months	Autologous peripheral blood	1.7 × 10^6^ × 3 (CD104^+^) 1.2 × 10^6^ × 3 (CD34^+^)	Injection	Subchondral drilling	- Improved WOMAC scores - Increased the case of avoidance of TKA	Treatments showed promise in disease modification with potential inhibition of OA progression.
Akgun et al. (2014)	*n* = 14	24 months	Autologous synovium	8 × 10^6^	Transplantation	Chondroplasty	- Improved KOOS pain, VAS-F, and Tegner scores - Improved MRI graft infill	Treatments effectively accelerate the recovery of chondral lesion of the knee.
Zhou et al. (2021)	*n* = 57	12 months	Autologous infrapatellar fat pad	3.91 × 10^6^	Injection	Chondroplasty	- Improved VAS rest, VAS motions, WOMAC total, and WOMAC function scores - Improved MOCART scores	The treatment provided an assistance in reducing pain and improving function of the knee.
Gupta et al. (2016)	*n* = 60	12 months	Allogeneic bone marrow	25 × 10^6^ 50 × 10^6^ 75 × 10^6^ 150 × 10^6^	Injection	None	- Improved VAS, WOMAC pain, and ICOAP total score in 25 and 50 million cell groups. - Not improved WORMS	A trend toward pain reduction was observed at the lowest cell dose of 25 million.
Vega et al. (2015)	*n* = 30	12 months	Allogeneic bone marrow	40 ×10^6^	Injection	None	- Improved VAS, WOMAC-pain, WOMAC-general, and LEQUESNE scores - Improved T2 relaxation time after treatments	Treatments provided clinically effective pain relief and improved the quality of cartilage.
Kuah et al. (2018)	*n* = 20	12 months	Allogeneic adipose	3.9 × 10^6^ 6.7 × 10^6^	Injection	None	- Improved VAS and WOMAC scores - No decrease in average lateral tibial cartilage volume in the 3.9M group	Pain reduced in intervention group, and lateral tibial cartilage loss was halted in the 3.9 M group, while the placebo group showed a significant cartilage loss.
Zhao et al. (2019)	*n* = 18	48 weeks	Allogeneic adipose	1.0 × 10^7^ × 2 2.0 × 10^7^ × 2 5.0 × 10^7^ × 2	Injection	None	- Improved WOMAC and SF-36 scores - Improved WORMS - Increased cartilage volumes	The treatment alleviated OA symptoms, and possible compositional changes of cartilage were suggested by quantitative MRI measurements.
Soltani et al. (2019)	*n* = 20	24 weeks	Allogeneic placenta	0.5~0.6 × 10^8^	Injection	None	- Improved VAS, KOOS and ROM - 10% of improved chondral thickness	The treatments provided clinical improvements.
Lim et al. (2021)	*n* = 89	60 months	Allogeneic umbilical cord blood	7.5 × 10^6^	Transplantation	Subchondral drilling	- Improved VAS, WOMAC, and IKDC scores in 60 months. - Improved cartilage grade in 48 weeks	UCB-MSC can be a viable regenerative treatment option.
Matas et al. (2018)	*n* = 26	12 months	Allogeneic umbilical cord	20 × 10^6^ 20 × 10^6^ × 2	Injection	None	- Improved WOMAC total score in the double injection group - Reduced VAS pain in the double injection group - No change in SF-36 pain - No difference in MRI scores	It was observed that repeated MSC treatment was superior to active comparator in knee OA.

## Data Availability

The data that support the findings of this study are available from the corresponding author upon reasonable request.

## Abbreviations

HTO: high tibial osteotomy; hG-CSF, human granulocyte colony-stimulating factor; DMEM, Dulbecco’s Modified Eagle’s Medium; HLA-DR, Human Leukocyte Antigen-DR isotype; PBS, phosphate-buffered saline; FBS, fetal bovine serum; α-MEM, Alpha Modification of Eagle’s Minimum Essential Media; WOMAC, Western Ontario and McMaster Universities Osteoarthritis; VAS, Visual Analog Scale; KOOS, Knee Injury and Osteoarthritis Outcome Score; ROM, Range of Motion; SF-36, Short Form 36; IKDC, International Knee Documentation Committee; TKA, Total Knee Arthroplasty; NPRS, Numeric Pain Rating Scale; WORMS, Whole-Organ Magnetic Resonance Imaging Score; MOCART, Magnetic Resonance Observation of Cartilage Repair Tissue; MRI, Magnetic Resonance Imaging; KL grade, Kellgren–Lawrence grade; MOAKS, MRI Osteoarthritis Knee Score; ICRS, International Cartilage Repair Society; BM, Bone Marrow; BMAC, Bone Marrow Aspirate Concentrate; HKA, Hip Knee Ankle; HTO, high tibial osteotomy; ICOAP, Intermittent and Constant Osteoarthritis Pain; ICRS, International Cartilage Repair Society; IKDC, International Knee Documentation Committee; KL grade, Kellgren–Lawrence grade; KOOS, Knee Injury and Osteoarthritis Outcome Score; MOAKS, MRI Osteoarthritis Knee Score; MOCART, Magnetic Resonance Observation of Cartilage Repair Tissue; MRI, magnetic resonance imaging; MSC, mesenchymal stem cells; NPRS, Numeric Pain Rating Scale; OA, osteoarthritis; PRP, platelet-rich plasma; ROM, range of motion; SF-36, Short Form 36; SVF, Stromal Vascular Fraction; TKA, Total Knee Arthroplasty; UCB, Umbilical Cord Blood; LIPUS, low-intensity pulsed ultrasound; ACL, anterior cruciate ligament; VAS, Visual Analogue Scale; WOMAC, Western Ontario and McMaster Universities Osteoarthritis; WORMS, Whole-Organ Magnetic Resonance Imaging Score.
